# Two Sides of the Same Quip: Humor Appeals Can Indirectly Reduce Reactance via Perceived Humor but Simultaneously Increase Reactance Independently of Perceived Humor

**DOI:** 10.3390/bs15111509

**Published:** 2025-11-07

**Authors:** Adam S. Richards, Nicholas S. Curcio, Sydney G. Hall

**Affiliations:** Department of Communication Studies, Furman University, Greenville, SC 29613, USA

**Keywords:** humor, psychological reactance, freedom threat, COVID-19, vaccination

## Abstract

This study assessed whether a humor appeal can reduce the degree to which freedom threatening language elicits psychological reactance and subsequently reduces positive vaccination attitude. A 2 (freedom threatening language: low vs. high) × 2 (humor appeal: absent vs. present) between-subjects experiment was conducted during the COVID-19 pandemic in the context of social media posts about vaccination using a sample of people eligible for vaccination (*N* = 190). Results showed that the humor appeal did not mitigate the effect of freedom threatening language on perceived threat to freedom, but it did separately reduce reactance via perceived humor. However, the presence of the humor appeal also directly contributed to reactance independently of how funny people found the appeal, ultimately reducing positive vaccination attitude. This study demonstrates how humor appeals have complex effects on resistance motivations that should be considered when using them as a persuasive strategy.

## 1. Introduction

If something is ridiculous, how can it be threatening?

(Francis et al., 1999, p. 171)

Despite being one of the deadliest pandemics in recorded human history (*The pandemic’s true death toll*, 2022), a significant number of Americans chose not to receive a COVID-19 vaccine. Various reasons account for such vaccine hesitancy, including misinformation about (Loomba et al., 2021), politicization of (Bolsen & Palm, 2022), and psychological reactance to social media posts about (Lu & Sun, 2022) the COVID-19 vaccine. This study focuses on the latter as a potential cause for COVID-19 vaccine hesitancy and how the use of humor appeals might mitigate such consequences. That is, we investigate whether humor functions as a message strategy that can reduce reactance to vaccine advocacy, which would otherwise contribute to a more negative attitude toward vaccination.

Psychological reactance is an aversive motivational state—conceptualized as a combination of negative cognitions and anger (Dillard & Shen, 2005)—that is brought about when people feel like their autonomy is threatened and causes them to act in ways to restore a sense of freedom (Brehm & Brehm, 1981). Persuasive appeals that cause people to experience reactance often elicit the very behaviors they intend to prevent (Burgoon et al., 2002). Several message strategies have been explored that attempt to reduce the degree to which freedom threatening language in an appeal ultimately manifests in reactance and unintended persuasive outcomes. These include freedom restoration postscripts (e.g., Bessarabova et al., 2013, 2017; Miller et al., 2007), inoculation forewarnings of reactance (e.g., Richards & Banas, 2015; Richards et al., 2017, 2021; Clayton et al., 2023), and empathy inductions (Shen, 2010, 2011), among others. In this vein, the use of humor has received some attention as a reactance mitigation strategy (e.g., Moyer-Gusé et al., 2018; Skalski et al., 2009).

But whether humor is useful for persuasion is debated (Meyer, 2000), and it is generally acknowledged that humor can lead to favorable or unfavorable outcomes depending on how appropriately it is employed in a given context (Gruner, 1985). Empirically, although humor appears to provide a minor benefit to persuasion (Walter et al., 2018), it can also reduce source credibility (Eisend, 2009) and have other negative consequences if not conveyed tactfully (Francis et al., 1999). Theoretically, humor has been shown to aid persuasion via a psychoemotive function by both reducing negative cognitions and negative affect in response to an appeal (Eisend, 2011). Given that psychological reactance is conceptualized as similarly psychoemotive (Dillard & Shen, 2005), perhaps the context of using humor to reduce reactance is advantageous.

Some research shows that humor reduces reactance because it distracts message recipients from making negative attributions (Strick et al., 2012; Young, 2008) or enhances positive affect (Skalski et al., 2009). Humor appeals have been shown to reduce reactance, particularly in vaccination contexts (Moyer-Gusé et al., 2018). Others showed that in the context of COVID-19, vaccination PSAs that lead with humor are less reactance-inducing among some audiences (Kim et al., 2024). Scholars have theorized that message recipients’ perceptions of humor serve as a mediating mechanism to account for reductions in reactance after exposure to health-related humor appeals (Betsch et al., 2020; Yuan & Lu, 2022).

Despite these studies showing a connection between humor and reactance, much of the fruitful work on reactance reduction strategies has demonstrated that such strategies function differently according to the intensity of the freedom threatening language also used in the persuasive appeal. That is, reactance reduction strategies can have different effects when paired with an appeal that is mild and suggestive versus forceful and dogmatic, with the latter typically being more reactance inducing according to meta-analyses (Li & Shi, 2025; Ma et al., 2025). For example, freedom restoration postscripts can reduce reactance, but mainly when freedom threatening language is high (Bessarabova et al., 2017). Inoculation can reduce reactance, but mainly when freedom threatening language is low (Richards et al., 2017). Non-red color cues can reduce reactance, but mainly when freedom threatening language is high (Armstrong et al., 2021). It is important to explore how reactance reduction strategies mitigate reactance in tandem with freedom threatening language, and the studies about reactance and humor mentioned in the previous paragraph neither manipulated nor measured perceptions of that variable. We do both here.

Given humor’s disarming effect on persuasive defenses (Strick et al., 2012) and message scrutiny (Young, 2008), we expect that messages that have higher freedom threatening language will elicit relatively less perceived freedom threat when paired with (versus than without) a humor appeal. That is, a joke will reduce the degree to which people perceive a freedom threatening appeal as freedom threatening. Similarly, given that freedom threatening language puts people on the defensive (Clayton, 2022) and elicits negative cognitions about the message (Li & Shi, 2025), we expect that a humor appeal is perceived as less humorous when paired with higher (versus than with lower) freedom threatening language. That is, a freedom threatening appeal will reduce the degree to which people perceive a joke as funny. Thus, we predict:

**H1:**  *The effect of freedom threatening language on perceived threat to freedom is weaker in the presence of a humor appeal compared to in the absence of a humor appeal.*

**H2:**  *The effect of a humor appeal on perceived humor is weaker in the presence of high freedom threatening language compared to low freedom threatening language.*

Further, we explore the nature of these effects in light of the multi-step reactance model. Quick et al. (2013) theorized the reactance process as one of serial mediation, in which message features—like freedom threatening language and humor appeals—first influence perceived threat to freedom, then reactance, and then persuasive outcomes like attitude. We expect the same here. In particular, we anticipate that the influence of message features is carried through to reactance via their associated perceived states. That is, freedom threatening language increases reactance by way of perceived threat to freedom (see Quick & Considine, 2008), and a humor appeal reduces reactance by way of perceived humor (see Yuan & Lu, 2022). This influence ultimately manifests in vaccination attitudes as an outcome that is endogenous to reactance. Thus, we predict:

**H3:**  *Perceived threat to freedom positively relates with state reactance.*

**H4:**  *Perceived humor negatively relates with state reactance.*

**H5:**  *Freedom threatening language reduces positive attitude toward vaccination indirectly via serial mediation of perceived threat to freedom and state reactance.*

**H6:**  *A humor appeal increases positive attitude toward vaccination indirectly via serial mediation of perceived humor and state reactance.*

**Figure 1:**
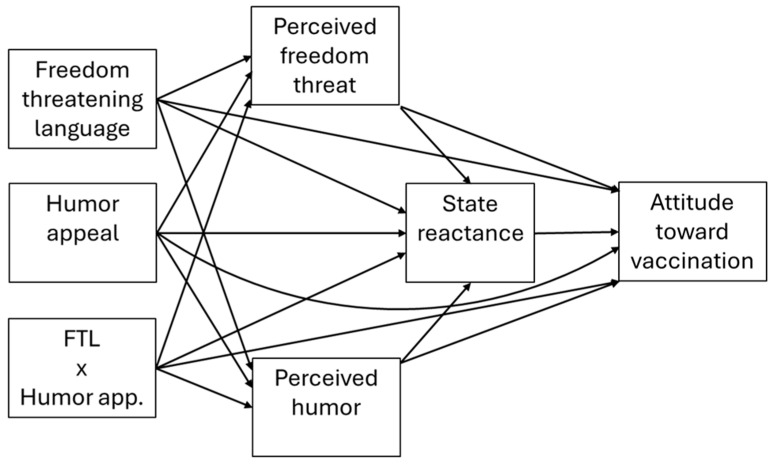
Serial mediation model.

These predictions are visually represented in the proposed model depicted in Figure 1. This serial mediation model also includes direct paths that account for the possibility of antecedent variables influencing persuasive outcomes via alternative routes from those theorized here. The partial mediation model reflects similar theorizing by Betsch et al. (2020), who argued that lower reactance results as a consequence of perceived funniness, and we also account for the role of perceived threat to freedom as an additional parallel mediator.

## 2. Materials and Methods

### 2.1. Participants

190 participants were recruited in the midst of the COVID-19 pandemic during April 2021 via emails to social networks and posts to social media. People qualified for participation if they were living in the USA and had not received the full sequence of a COVID-19 vaccine. Of the final sample, 82 (43%) had not received any dose of a vaccine and 108 had received only one dose of a two-dose vaccine sequence. Most identified as female (54%) or male (44%), with 1% reporting as non-binary or third gender, and 0.5% choosing not to identify. Ages ranged from 18 to 59 (*M* = 24.41, *SD* = 8.81). Most identified as White (85%), followed by Black or African American (10%), Asian (5%), or Native Hawaiian or Pacific Islander (0.5%), with 6% separately identifying as Hispanic. Most reported completing some college (59%), graduating from high school (16%), or earning a college (16%), associate (4%), or graduate (3%) degree.

### 2.2. Design and Procedures

All procedures received institutional review board approval. The study took place online via the Qualtrics (Waltham, MA, USA) platform. The experiment used a 2 (freedom threatening language: low vs. high) × 2 (humor appeal: absent vs. present) between-subjects design. After providing informed consent, participants were randomly assigned automatically by Qualtrics to view an alleged Twitter post from the Centers for Disease Control and Prevention (CDC; Atlanta, GA, USA) that promoted COVID-19 vaccination. Participants then completed a post-test survey and were debriefed. No attention checks were utilized.

### 2.3. Experimental Materials

Manipulations took the form of a screen capture of a three-sentence Twitter post attributed to the CDC, a medium and source used in previous research relating to humor appeals in the context of COVID-19 (Vaala et al., 2022). In one sentence, freedom threatening language was manipulated by altering the degree to which the directive was mild and polite (i.e., “Please choose to get a COVID-19 vaccine when you are eligible.”) or forceful and dogmatic (i.e., “You MUST get a COVID-19 vaccine as soon as you are eligible!”). In another sentence, a humor appeal was manipulated by including a neutral statement about the efficacy of the vaccine (i.e., “Every vaccine approved by the FDA is highly effective at protecting against COVID-19.”) or a joke attempting to minimize concerns about the vaccine (i.e., “If you survived the arcade ball pit as a kid, you have no need to worry about what is in the vaccine.”).^1^ A third sentence, which was consistent across conditions, concluded the post by directing readers to a link for more information about the vaccine.

### 2.4. Measures

Unless otherwise noted, all composite variables were formed by taking an average of the measured items. Factor analyses indicated that all scales were unidimensional.

#### 2.4.1. Perceived Threat to Freedom

Perceived freedom threat was assessed on a seven-point Likert-type scale via four items taken from Dillard and Shen (2005; e.g., “The message threatened my freedom to choose.”; *M* = 3.54, *SD* = 1.48 α = .85).

#### 2.4.2. Perceived Humor

Three items measured perceived humor using a seven-point semantic differential scale taken from Bippus et al. (2012; i.e., “I found this message to be: not funny/funny, not humorous/humorous, not amusing/amusing.”; *M* = 3.01, *SD* = 2.03, α = .98).

#### 2.4.3. State Reactance

We used a seven-point Likert-type scale to measure felt anger with four items from Dillard and Shen (2005; e.g., “While viewing this message, I felt angry.”; *M* = 2.78, *SD* = 1.57, α = .96) and negative cognitions with three items from Quick et al. (2015; e.g., “The thoughts I had about this message were negative.”; *M* = 3.12., *SD* = 1.55, α = .95). Following the procedures used by Shen (2010) and others (e.g., Bünzli et al., 2025; Clayton, 2022; Kriss et al., 2022; Richards et al., 2022), these two measures were each standardized and averaged to form a composite measure of reactance.

#### 2.4.4. Attitude

The degree to which COVID-19 vaccines were perceived positively was assessed with five-items using a seven-point semantic differential scale taken from McCroskey and Richmond (1989; e.g., “To me, COVID-19 vaccines are bad/good, negative/positive, foolish/wise.”; *M* = 5.98, *SD* = 1.41, α = .97).

## 3. Results

The model presented in Figure 1 was assessed using PROCESS 3.5 (Model 80; Hayes, 2018) with 95% confidence intervals and 1000 bootstrap samples. A variable representing the order with which the freedom threatening language and humor appeal manipulations were presented was covaried to reflect that aspect of the design.^2^

We first assessed the effect of the experimental variables and their interaction on perceived threat to freedom and perceived humor. These models also served as manipulation checks by assessing the significance of the effect of the induction on the corresponding perceived outcome. The first hypothesis predicted that the presence of a humor appeal would mitigate the effect of freedom threatening language on perceived threat to freedom. As seen in Table 1, freedom threatening language had a significant main effect on perceived threat to freedom (*b* = 0.94, *p* = .001). People perceived less freedom threat in the low freedom threatening language condition (*M* = 3.09, *SD* = 1.45) compared to the high condition (*M* = 3.97, *SD* = 1.38), indicating a successful manipulation. However, there was no evidence that a humor appeal moderated the nature of this effect (b = −0.12, *p* = .77). Therefore, humor did not mitigate the effect of freedom threatening language on perceived threat to freedom. *H1* was not supported.

**Figure 2:**
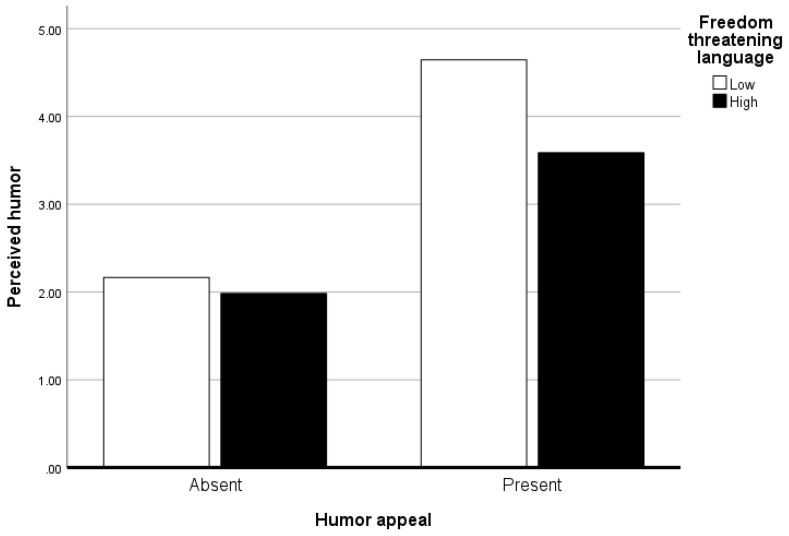
Perceived humor as a function of humor appeal and freedom threatening language.

The second hypothesis predicted that the presence of freedom threatening language would mitigate the effect of a humor appeal on perceived humor. As seen in Table 1, the humor appeal had a significant main effect on perceived humor (*b* = 2.48, *p* < .001). People perceived more humor in the presence of the humor appeal (*M* = 4.08, *SD* = 2.08) compared to in its absence (*M* = 2.08, *SD* = 1.46), indicating a successful manipulation. Additionally, there was a marginally significant interaction between the two experimental variables (*b* = −0.88, *p* = .08) which is visually demonstrated in Figure 2. The significant conditional effects indicated that the effect of the humor appeal was more robust when freedom threatening language was low (*b* = 2.48, *SE* = 0.36, *t* = 6.81, *CI*[1.76, 3.20]) rather than high (*b* = 1.60, *SE* = 0.36, *t* = 4.49, *CI*[0.90, 2.31]). Given the marginally significant interaction but significant conditional effects, we take this as tentative support for *H2*.

We also predicted that perceived freedom threat would lead to more reactance (*H3*) and perceived humor would lead to less reactance (*H4*). Results in Table 2 show that both of these hypotheses were supported. State reactance associated positively with perceived threat to freedom (*b* = 0.47, *p* < .001) and associated negatively with perceived humor (*b* = −0.07, *p* = .01). Thus, people who perceived the message as more autonomy threatening were more reactant and those who perceived the message as funnier were less reactant. Further, the humor appeal induction exhibited a positive main effect on state reactance (*b* = 0.33, *p* = .03). Here, people exposed to the humor appeal reported more, not less, state reactance. That is, those who read the joke became more reactant independently of how funny they perceived the appeal.

Assessment of indirect effects also indicated that the message inductions affected reactance by way of their associated perceived states. That is, freedom threatening language significantly increased reactance via perceived threat to freedom (*b* = 0.44, *SE_boot_* = 0.13, *CI_boot_*[0.17, 0.68]). Similarly, the humor appeal significantly reduced reactance via perceived humor (*b* = −0.17, *SE_boot_* = 0.08, *CI_boot_*[−0.34, −0.02]).

Finally, we regressed attitude toward vaccination on the aforementioned variables as predictors. Table 3 shows that only state reactance exhibited a direct effect on attitude (*b* = −0.54, *p* < .001). Assessment of indirect effects showed that the experimental manipulations also influenced attitude toward vaccination via various routes of serial mediation. As expected, freedom threatening language reduced positive attitude toward vaccination via the path of freedom threatening language → perceived threat to freedom → reactance → attitude (*b* = −0.24, *SE_boot_* = 0.11, *CI_boot_*[−0.48, −0.08]), supporting *H5*. Also as expected, the humor appeal increased positive attitude toward vaccination via the path of humor appeal → perceived humor → reactance → attitude (*b* = 0.09, *SE_boot_* = 0.05, *CI_boot_*[0.01, 0.20]), supporting *H6*. Additionally, the humor appeal also reduced positive attitude toward vaccination via the path of humor appeal → reactance → attitude (*b* = −0.16, *SE_boot_* = 0.09, *CI_boot_*[−0.35, −0.003]). In the latter case, people exposed to the humor appeal had a more negative attitude toward vaccination by way of reactance, independently of how funny they perceived the appeal.

## 4. Discussion

The purpose of this study was to test whether humor can mitigate freedom threatening language that would otherwise induce psychological reactance. We found that freedom threatening language increased reactance and led to subsequent counterattitudinal change via perceived threat to freedom regardless of whether a humor appeal was present. This finding replicates the robust research base showing that threatening message features lead to more reactance and subsequent counterpersuasive outcomes (Li & Shi, 2025; Ma et al., 2025). In short, the presence of a humor appeal did not alter how freedom threatening language translated into perceived freedom threat and reactance. One possible explanation for this finding is that freedom threatening language functioned as the more prominent message feature compared to the joke itself, so the presence of humor was not a salient enough cue to alter how freedom threat was perceived. Further, we found that a humor appeal reduced reactance, and led to subsequent proattitudinal change, via perceived humor regardless of whether freedom threatening language was low or high. In this sense, people who found humor appeals to be funnier were less likely to be triggered into a reactance response. This finding replicates other work about the role of perceived humor in reducing reactance (Yuan & Lu, 2022).

We expected that the message features of a humor appeal and freedom threatening language would combine to ultimately affect reactance. In particular, we predicted that, in the presence of a humor appeal, increasingly freedom threatening language would be perceived as less freedom threatening, thereby eliciting less reactance. That is, humor would buffer the negative consequences of freedom threatening language. This was not the case. Instead of the humor appeal conditioning the effects of freedom threatening language, we found that the two message features essentially operated as separate persuasive cues that independently affected reactance via distinct psychological mechanisms.

However, we also found that the presence of a humor appeal directly contributed to more reactance and negative attitude change through a route that bypassed perceptions of humor and perceptions of freedom threat. That is, the presence of a humor appeal led people to experience more anger and negative cognitions in ways not attributable to the theorized mediating mechanisms in our model. This finding is similar to other research (Betsch et al., 2020) that found a nearly significant (i.e., *p* = .06) positive direct effect of a humor induction on reactance despite perceived humor having no mediating effect. Notably, the direct effect of the humor appeal in our study was nearly twice as strong as the opposing indirect effect of the humor appeal via humor perceptions. When considered in tandem, these findings therefore suggest that the inclusion of a humor appeal functioned as a net detriment when it came to preventing reactance.

On the one hand, this study supports the notion that a joke that is found to be funny by message recipients can serve as a strategic mechanism for reducing motivations that lead to persuasive resistance. On the other hand, our findings suggest that the inclusion of a humor appeal in a persuasive message can also reduce the message’s ability to persuade due to reasons outside of a joke being thought of as humorous. In this study, we suspect that the joke may have undermined the message source’s credibility (see Eisend, 2009) or led to discounting of the message (see Nabi et al., 2007) by causing people to see the CDC’s statement as flippant, unserious, and disrespectful to vaccine skeptics—even while still being perceived as funny—which ultimately reduced its persuasiveness. Some research showed that sources of COVID-19 information on social media who used humor enhanced audience attributions of credibility only when the crisis stage of the pandemic was less, rather than more, severe (Xiao & Yu, 2022). Given that our study was conducted when the vaccine was just becoming publicly available and infection rates were high, it is plausible to assume that the crisis stage was actually more, rather than less, severe. Other research showed that among some message recipients, the CDC was deemed to be a less believable source of information about COVID-19 vaccination when their messages conveyed humor compared to when they did not (Yoon et al., 2023). In this context, humor may have served a divisive function by differentiating the CDC from message recipients (see Meyer, 2000). Thus, although humor scholars have argued that humor can either be helpful *or* harmful to persuasion depending on its appropriate use in different contexts (Francis et al., 1999), this study showed that humor can be helpful *and* harmful in the same context by both reducing and increasing reactance through separate psychological mechanisms. Alongside the role of perceived funniness in reducing persuasive resistance, future research should assess whether source evaluations function as one of these possible mechanisms to account for how a humor appeal can simultaneously enhance resistance.

These findings also point to questions raised by others regarding the theoretical nature of the state reactance variable. Reactance scholars theorize that the variable of intertwined anger and negative cognitions ought to only be construed as reactance if it is caused by perceptions of freedom threat (Quick et al., 2013). However, others acknowledge that this motivational state can be brought about via other psychological mechanisms and might better be construed as a more general concept representing persuasive resistance (Ratcliff, 2021). In our model, one of the antecedents of the intertwined variable was perceived threat to freedom, aligning with the traditional perspective of reactance theorists (Quick et al., 2013). However, independent of perceived freedom threat, antecedents of the intertwined variable also included perceived humor and the humor appeal manipulation itself. These multiple causes suggest that the intertwined variable partially captures autonomy-threatened reactance but also represents other forms of persuasive resistance brought about by alternative psychological mechanisms besides perceived freedom threat. Further work should aim to disentangle the complexities of when intertwined anger and negative cognitions qualify as a measure of state reactance and when it does not.

Practically, this research suggests that a humor appeal can successfully elicit humor and influence attitudes as intended but still have other adverse consequences to persuasion that outweigh its benefit. Thus, one implication for message designers is to be wary of employing humor appeals in persuasive messaging unless they are fully aware of the potential side effects of the joke when audiences make attributions of message appropriateness, source credibility, information quality, etc. In cases where humor is employed, we found no evidence to suggest that jokes produce different outcomes when paired with high versus low freedom threatening language. Accordingly, persuasive sources may be able to use similar humor appeals with messages that are mild and suggestive as well as those that are forceful and dogmatic, although the former use of language is less likely to induce reactance.

As with all research, this study had limitations. For one, this study used only one humor appeal, and the results may be limited to the nature of the specific joke communicated here. Although the findings are important because they demonstrate a reason for caution when employing humor appeals, it remains to be known whether different forms of humor would lead to the same conclusions. Similarly, this study was in the context of COVID-19 vaccination, and the results may be unique to this health context. In addition, the convenience sample of online participants reduces the degree to which these results generalize to a representative group of Americans. Further, our sample lacked sufficient power to detect small interaction effects, so the absence of significant moderation between humor and freedom threatening language may be an artifact of the sample size. Finally, all measurements occurred in the posttest survey, so any directional associations among measured variables are theoretically, rather than methodologically, imposed.

This experiment demonstrated that a social media post advocating for the COVID-19 vaccine induced greater psychological reactance when it possessed intense freedom threatening language. If the post also conveyed a humor appeal, it reduced reactance via perceived humorousness. But, the humor appeal directly increased reactance independently of perceived humorousness. The study found that a humor appeal did not make freedom threatening language any more or less freedom threatening, but it can lead to less (via the mediating effect of perceived humor) and more (via a direct effect) persuasive resistance. Thus, humor can indeed function as a double-edged sword (see Meyer, 2000).

## Figures and Tables

**Table 1:** Predictors of perceived threat to freedom and perceived humor.

	Perceived Freedom Threat					Perceived Humor				
Effect	Estimate	*SE*	95% CI		*p*	Estimate	*SE*	95% CI		*p*
			*LL*	*UL*				*LL*	*UL*	
FTL ^a^	0.94	0.28	0.39	10.50	.001	−0.18	0.35	−0.87	0.50	.60
Humor appeal ^b^	0.01	0.30	−0.58	0.59	.99	2.48	0.36	1.76	3.20	.000
FTL × Humor appeal	−0.12	0.41	−0.94	0.70	.77	−0.88	0.51	−0.188	0.13	.08
Order ^c^	0.00	0.21	−0.41	0.41	.99	0.18	0.26	−0.32	0.68	.48

*Note.* FTL = freedom threatening language. CI = confidence interval; *LL* = lower limit; *UL* = upper limit. ^a^ 0 = low, 1 = high. ^b^ 0 = absent, 1 = present. ^c^ 0 = FTL first, 1 = humor appeal first.

**Table 2:** Predictors of state reactance.

Effect	Estimate	*SE*	95% CI		*p*
			*LL*	*UL*	
Perceived freedom threat	0.47	0.03	0.40	0.53	.000
Perceived humor	−0.07	0.03	−0.25	−0.01	.01
FTL ^a^	0.01	0.13	−0.25	0.27	.93
Humor appeal ^b^	0.33	0.15	0.03	0.63	.03
FTL × Humor appeal	−0.28	0.19	−0.65	0.10	.14
Order ^c^	−0.10	0.09	−0.29	0.08	.27

*Note*. FTL = freedom threatening language. CI = confidence interval; LL = lower limit; UL = upper limit. ^a^ 0 = low, 1 = high. ^b^ 0 = absent, 1 = present. ^c^ 0 = FTL first, 1 = humor appeal first.

**Table 3:** Predictors of positive vaccination attitude.

Effect	Estimate	*SE*	95% CI		*p*
			*LL*	*UL*	
State reactance	−0.54	0.14	−0.82	−0.26	.000
Perceived freedom threat	−0.15	0.09	−0.34	0.03	.10
Perceived humor	−0.01	0.05	−0.12	0.09	.82
FTL ^a^	0.08	0.25	−0.42	0.59	.74
Humor appeal ^b^	0.52	0.30	−0.07	10.11	.09
FTL × Humor appeal	−0.18	0.37	−0.92	0.58	.63
Order ^c^	0.13	0.18	−0.24	0.49	.50

Note. FTL = freedom threatening language. CI = confidence interval; LL = lower limit; UL = upper limit. ^a^ 0 = low, 1 = high. ^b^ 0 = absent, 1 = present. ^c^ 0 = FTL first, 1 = humor appeal first.

## Data Availability

The data presented in this study are available on request from the corresponding author.

## Notes

1 Given prior research about order effects of humor and risk information on reactance (Kim et al., 2024), the order of the freedom threatening language and humor appeal manipulation was counterbalanced randomly to offset potential order effects of the two manipulations. This variable did not demonstrate any significant main effects, nor did it interact with the experimental variables to predict any dependent variables—meaning order effects did not occur in this study—but we controlled for order in our analyses given its role in the study’s design. Substantive results were meaningfully equivalent regardless of whether this variable was entered as a covariate.

2 Results reported do not include vaccination status (i.e., no dose vs. one dose of a two-dose sequence) as a covariate given that this variable was balanced via random assignment procedures. However, results were meaningfully equivalent in models that included vaccination status as a covariate.

## Abbreviations

The following abbreviations are used in this manuscript:

CDC	Centers of Disease Control and Prevention
FTL	Freedom threatening language
